# Full-Length Transcriptome Analysis of the Halophyte *Nitraria sibirica* Pall

**DOI:** 10.3390/genes13040661

**Published:** 2022-04-08

**Authors:** Huilong Zhang, Zhen Liu, Aishuang Hu, Haiwen Wu, Jianfeng Zhu, Fengzhi Wang, Pingping Cao, Xiuyan Yang, Huaxin Zhang

**Affiliations:** 1 Institute of Ecological Protection and Restoration, Chinese Academy of Forestry, Beijing 100091, China; hlzhang@caf.ac.cn (H.Z.); hash0207@163.com (A.H.); 13621143840@163.com (H.W.); jfzhu@caf.ac.cn (J.Z.); yangxiuyan@caf.ac.cn (X.Y.); 2 The Comprehensive Experimental Center of Chinese Academy of Forestry in Yellow River Delta, Dongying 257000, China; 3 Hebei Key Laboratory of Crop Salt-Alkali Stress Tolerance Evaluation and Genetic Improvement, Cangzhou 061001, China; liuzhen84575151@163.com (Z.L.); cznky420@126.com (F.W.); cp0820@126.com (P.C.); 4 Academy of Agriculture and Forestry Sciences, Cangzhou 061001, China; 5 Institute of Coastal Agriculture, Hebei Academy of Agriculture and Forestry Sciences, Tangshan 063299, China

**Keywords:** full-length transcriptome analysis, *Nitraria sibirica*, H^+^-PPase, salt stress

## Abstract

Background: *Nitraria sibirica* Pall. is one of the pioneer tree species in saline–alkali areas due to its extreme salt tolerance. However, the lack of information on its genome limits the further exploration of the molecular mechanisms in *N. sibirica* under salt stress. Methods: In this study, we used single-molecule real-time (SMRT) technology based on the PacBio Iso-Seq platform to obtain transcriptome data from *N. sibirica* under salt treatment for the first time, which is helpful for our in-depth analysis of the salt tolerance and molecular characteristics of *N. sibirica*. Results: Our results suggested that a total of 234,508 circular consensus sequences (CCSs) with a mean read length of 2121 bp were obtained from the 19.26 Gb raw data. Furthermore, based on transcript cluster analysis, 93,713 consensus isoforms were obtained, including 92,116 high-quality isoforms. After removing redundant sequences, 49,240 non-redundant transcripts were obtained from high-quality isoforms. A total of 37,261 SSRs, 1816 LncRNAs and 47,314 CDSs, of which 40,160 carried complete ORFs, were obtained. Based on our transcriptome data, we also analyzed the coding genes of H^+^-PPase, and the results of both bioinformatics and functional analyses indicated that the gene prediction via full-length transcripts obtained by SMRT technology is reliable and effective. In summary, our research data obtained by SMRT technology provides more reliable and accurate information for the further analysis of the regulatory network and molecular mechanism of *N. sibirica* under salt stress.

## 1. Introduction

*N. sibirica* is a typical salt-diluting halophyte which can complete its life cycle in desert and saline–alkali soils. Therefore, *N. sibirica* is considered to be the pioneer tree for afforestation in saline–alkali areas, and it is widely used in the landscaping and bioremediation of saline–alkali land [1]. In addition, *N. sibirica* can also be used as an economic plant in saline–alkali areas for its medicinal value and edible fruit [2,3]. To date, some studies have tried to reveal the physiological basis of salt tolerance in *N. sibirica* under salt stress [1,4,5,6]. However, due to the lack of a comprehensive and robust reference genome, most of these studies focused on physiological analyses, such as the accumulation of organic osmolytes [6], distribution of Na^+^ and K^+^ in various tissues [1,4,5] and changes in antioxidant enzyme activities [7].

Transcriptome data obtained based on high-throughput sequencing technology can reflect the gene types and expression changes in cells, which contributes to an overall, big-data-level analysis which can better analyze gene expression and regulatory networks [8,9]. High-throughput RNA sequencing can be carried out without relying on the reference genome, so this technology can be used for the analysis of most species [8,10,11,12]. So far, there has been a large amount of research based on second-generation sequencing technology. Some studies used the transcriptome technique to reveal the molecular regulation mechanism of *N. sibirica* under salt stress [13]. The massive data obtained using the second-generation sequencing technology laid the foundation for uncovering the regulatory mechanism for salt tolerance in *N. sibirica*. However, because of the constraints of the second-generation sequencing platform on the read length of the sequence, the RNA needs to be broken into short fragments (<500 bp) [14,15] for sequencing, and finally the transcripts are acquired by short read assembly. The transcripts obtained in this way are likely to be unsuccessfully or incompletely assembled [14,16,17]. 

Single-molecule real-time (SMRT) sequencing, such as that using the PacBio sequencing platform (PacBio, Menlo Park, CA, USA), is a third-generation sequencing technology which can effectively overcome the disadvantages of second-generation sequencing [14,16,18]. SMRT sequencing technology allows the direct reading of cDNA and obtains high-quality, complete and accurate reads [16]. Based on this technique, the average reading length range is from 10 kb to 15 kb and the maximum length can reach 70 kb, which makes it easier to identify the isomers of shared exons and does not require assembly [14,16,19,20]. In addition, the third-generation sequencing technology can also identify and obtain new transcripts or genes and can be used to supplement genome annotation information, which helps to accurately analyze more information, such as that found in homologous genes and superfamily genes. To date, based on the advantages of the third-generation sequencing technology via the PacBio sequencing platform, this technology has been successfully applied to some species, such as humans [20], red clover (*Trifolium pratense*) [21], Purslane (*Portulaca oleracea*) [17], *Ananas comosus* var *bracteatus* [22] and pear (*Pyrus betulifolia* Bunge) [23]. However, full-length transcript data based on SMRT sequencing technology has not been reported in *N. sibirica*.

In our previous study, we found that the growth of *N. sibirica* was promoted under 200 mM NaCl concentration [1,4] but decreased under higher than 300 mM or without NaCl treatment. This is a response specific to salt stress for most halophytes, most of which grow best in low salt concentrations. In other words, halophytes are uniquely adapted to salt environments. To better explain this characteristic of *N. sibirica*, 200 mM NaCl treatment was used for sequencing in this study. In the present study, we obtained the full-length transcriptome data of *N. sibirica* through SMRT sequencing via the PacBio sequencing platform. Based on the high-quality and accurate transcriptome data, CDS (sequence coding for amino acids in protein) identification, simple sequence repeat (SSR) prediction, long-chain noncoding RNA (LncRNA) analysis and function annotation were performed. Meanwhile, we identified coding genes of H^+^-PPase based on full-length transcriptome data, analyzed their subcellular localization information and verified their expression in yeast using the DUAL membrane system. This study provides full-length transcript data to support the molecular and gene-function studies of *N. sibirica*, and also lays the foundation for the further clarification of the regulatory mechanism of *N. sibirica* under salt stress.

## 2. Materials and Methods

### 2.1. Plant Material and Salt Treatment

The seeds of *N. sibirica* were collected from saline–alkaline beach of Keluke lake in Qaidam Basin of Qinghai province, China. The seeds were treated in ddH_2_O for 24 h at 40–50 °C, then buried in wet sand and placed in the conservatory for germination. After germination, seedlings were transferred to a basin filled with a vermiculite:perlite mixture (3:1 w:w) and cultured in the greenhouse of the Chinese Academy of Forestry at 28/22 °C day/night and at 60–80% relative humidity. After 9 weeks of culture, plants with similar growth were selected for 200 mM NaCl treatment. The roots, stems and leaves were harvested and frozen in liquid N_2_ and stored at −80 °C for RNA extraction.

### 2.2. Nucleic Acid Extraction and SMRT Sequencing

An RNA extraction kit (Takara, Kusatsu, Japan) was used to obtain the total RNA, and all samples were mixed. The Qubit Fluorometer was used to assess the concentration of RNA under the processing of the Qubit RNA IQ Assay Kit. The integrity of the RNA was assessed based on data measured using the 2100 Agilent Bioanalyzer system (Agilent Technologies, Santa Clara, CA, USA). Library construction can only be performed when the mass of RNA meets a 280/260 ratio of 1.9 to 2.1, a 230/260 ratio of 2.0 to 2.4 and a RIN value greater than 7.0. The library for this experiment was made by mixing equal amounts of total RNA extracted from three different tissues.

SMRTbell library was prepared and sequenced following the process of the PacBio Sequel platform (Pacific Biosciences, CA, USA). The cDNA fragments were obtained via the PCR process with oligo dT as a primer and screened according to length (two sizes, <4000 bp and >4000 bp). Then, after a series of modifications, the SMRT adaptor was connected to complete the library construction. Finally, the PacBio Sequel SMRT sequencer from Pacific Biosciences was used to obtain the raw data.

### 2.3. Preprocessing of Raw Data

SMRTlink 6.0 software was used to analyze the raw data and filter out all polymerase reads of less than 50 bp with a quality less than 0.90 to obtain the clean reads. A circular consensus sequence (CCS) was obtained from subread BAM files with min FullPass = 3 and min Predicted Accuracy = 0.9, CCS.BAM files were output. Furthermore, the BAM files were segmented into both full-length (FL) and non-full-length non-chimeric (FLNC) based on pbclassify. The FL and FLNC transcripts were identified through traces of the poly(A)-tail signal and the 5′ and 3′ cDNA primers in error-corrected reads of insert (ROI). The consistency sequences were obtained by iterative error correction (ICE) approximation clustering, and the FL consistency sequences in ICE were polished using Quiver. We selected the consensus data with a post-correction accuracy of >0.99 for subsequent analysis. The completeness of the transcriptome was assessed via BUSCO/v3.0.2 with the embryophyta_odb9 database [24].

### 2.4. CDS, SSR, Transcription Factors (TFs), LncRNA Analysis

The coding sequences were predicted by TransDecoder (https://github.com/TransDecoder/TransDecoder/releases, accessed on 31 August 2020), and the SSR identification was performed based on the MISA database (https://webblast.ipk-gatersleben.de/misa/, accessed on 31 August 2020). Transcription factors were predicted using ITAK software. LncRNA candidate sequences were screened from the putative protein-coding RNAs in the transcripts according to bricks with lengths of more than 200 nt. The obtained transcripts were further confirmed according to the CPC/CNCI/CPAT/Pfam database for their ability to distinguish protein-coding genes from non-coding genes [25].

### 2.5. Functional Annotation

The non-redundant transcript function information was obtained through BLASTx (version 2.2.26) [26] with an e-value < 10^−5^ based on the following databases: NCBI non-redundant protein sequences database (NR), Protein family (Pfam), a manually annotated and reviewed protein sequence database (Swiss-Prot), Gene Ontology Consortium (GO), Cluster of Orthologous Groups of proteins (COG), Clusters of Orthologous Groups of proteins (KOG), evolutionary genealogy of genes: Non-supervised Orthologous Groups (eggNOG) and Kyoto Encyclopedia of Genes and Genomes (KEGG) [25].

### 2.6. Alternative Splice Detected

To obtain alternative splicing (AS) events for *N. sibirica*, BLAST with high-intensity settings was performed directly using Iso-SeqTM data. The screening of products for candidate AS events was based on the following criteria. First, two high-scoring segment pairs (HSPs) must exist in the sequence and have the same direction. There is at least one sequence that should be contiguous or have a small “overlap” size (less than 5 bp) in the same alignment. In addition, the “AS Gap” appears in another sequence, and consecutive sequences should be almost completely aligned with distinct sequences. We defined a sequence with a gap that should be greater than 100 basis points and at least 100 basis points from the 3′/5′ end as an AS Gap.

### 2.7. Phylogenetic and Subcellular Localization Analysis

MEGA-X software (Temple, Philadelphia, PA, USA) was used to reconstruct the phylogenetic tree with the neighbor-joining method, and the bootstrap value was set to 1000. TMHMM (http://www.cbs.dtu.dk/services/TMHMM/, accessed on 31 August 2020) was used for the transmembrane domain analysis of the screened candidate genes.

### 2.8. Function Analysis of H^+^-PPase

RNA was obtained from *N. sibirica* following the manufacturer’s protocol for the E.Z.N.A. Total RNA Kit (Omega Bio-Tek, Doraville, GA, USA). The first-strand cDNA was obtained from 1 μg of total RNA via HiFiScript gDNA Removal RT MasterMix (CoWin Biosciences, Beijing, China). The complete coding sequencing was amplified with the specific primers shown in Supplementary Table S1.

Function assays were performed following the DUAL membrane starter kit user Manual (Dualsystems Biotech AG, Schlieren, Switzerland). The coding sequences of two H^+^-PPase genes were obtained via PCR amplification, and the specific primers for the target genes are listed in Supplementary Table S1. The vectors were cotransformed into yeast strain NMY51 according to the standard process. The empty vectors were used as negative controls. Function was analyzed based on their ability to grow on Leu/Trp/His/Ade+X-α-Gal plat.

## 3. Results

### 3.1. The Sequencing Data Analysis of N. sibirica

**Figure 1:**
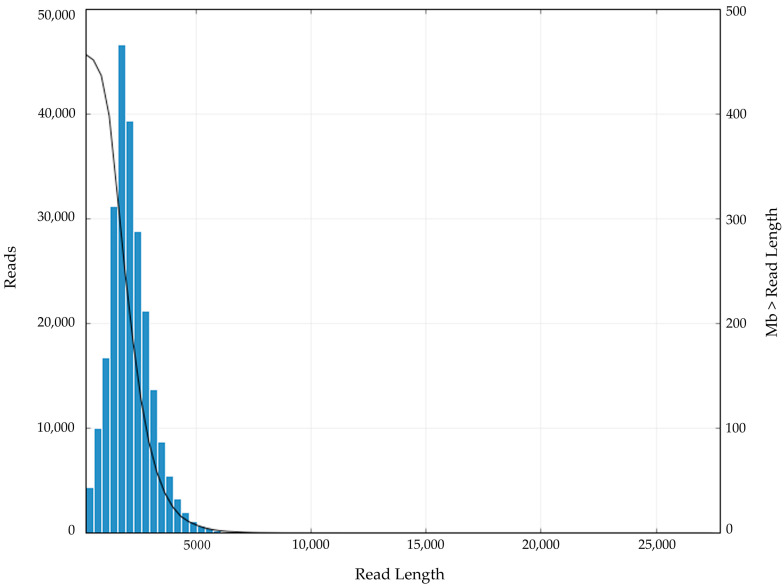
Distribution of the number and length of raw reads.

The qualified library was sequenced using SMRT, and the redundant or error consensus sequences were removed from the original sequences. A total of 19.26 Gb raw data were identified. The raw reads were mainly distributed in the range from 1 to 5000 bp, and the mean length was 2080 bp (Figure 1).

**Figure 2:**
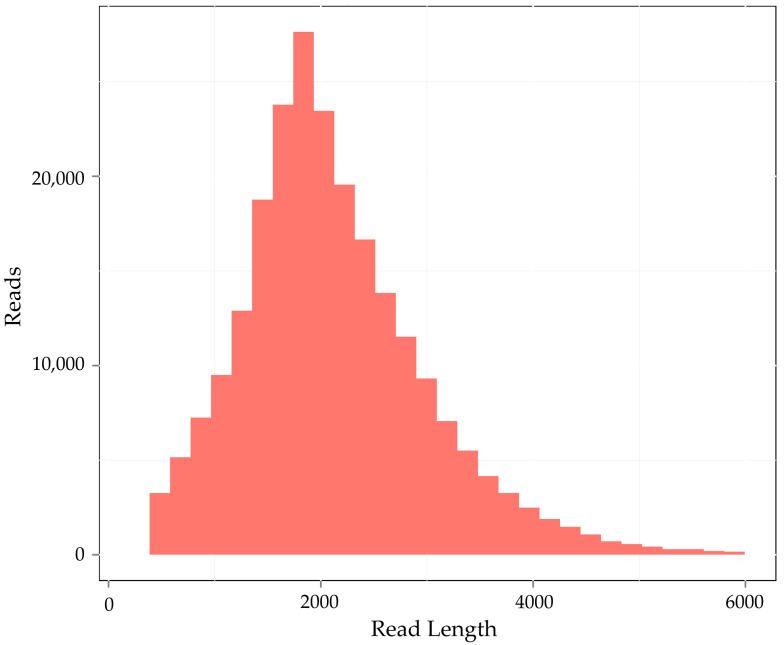
Distribution of the read number and length of CCS.

**Figure 3:**
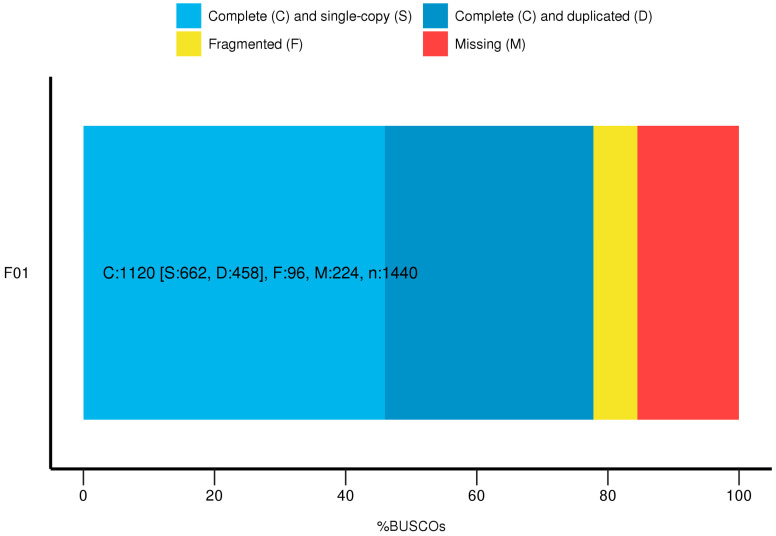
Result of the BUSCO assessment.

In order to further reduce the error rate, we carried out circular consensus sequence (CCS) analysis to obtain accurate data. In the present study, we obtained a total of 234,508 CCSs based on the full-length transcriptome data, with an average length of 2121 bp. In addition, we also constructed a consensus-length distribution map to show the calculation results based on the consensus length and found that the consensus sequence was dominated by a length of 1–4000 bp (Figure 2). The number of full-length non-chimeric (FLNC) readings was 195,027, accounting for 83.16%, of which the number of high-quality consensus isomers was 92,116. In order to further remove the redundant sequences, we used the CD-HIT program to filter them and finally obtained 49,240 transcripts for subsequent analysis. The analysis of transcriptome completeness with BUSCO is shown in Figure 3. Using the 1440 BUSCO plant set (embryophyta_odb9 database), a total of 1120 (77%) genes from the transcriptome were complete, with 662 (46%) single-copy genes and 458 (34.8%) duplicated; 96 (6.7%) were fragmented and 224 (15.5%) were missing BUSCOs (Figure 3). 

### 3.2. Prediction of Coding Sequences

**Figure 4:**
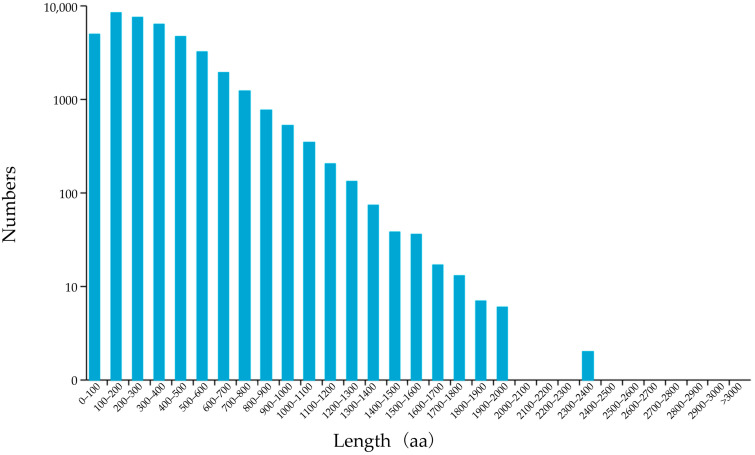
The predicted length distribution of the protein encoded by the complete ORF.

We used TransDecoder sequencing data to screen the coding sequence. A total of 47,314 coding sequences were predicted (Supplementary Table S3), of which 40,160 coding sequences had initial and termination codons, and these sequences were defined as a complete open reading frame (ORF). The predicted length distribution of the protein encoded by the complete ORF region is shown in Figure 4, in which amino–acid sequence lengths ≤ 1000 aa accounted for 97.81% (39,287), followed by those from 1000 to 2000 aa, which accounted for 2.15% (867), and for those >2000 aa, there were only six.

**Figure 5:**
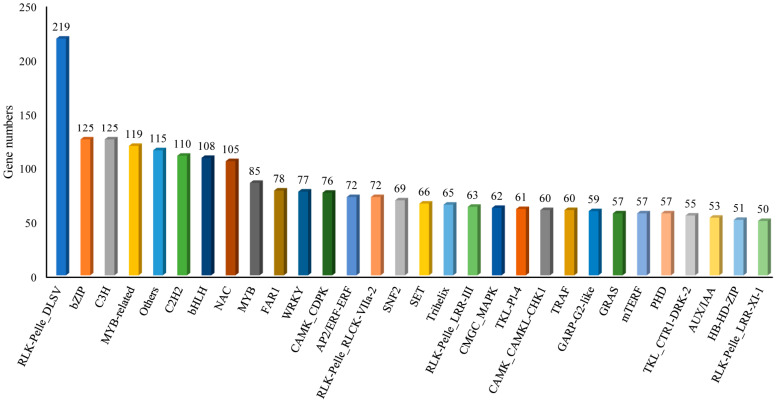
Numbers and families of the top 30 TFs in *N. sibirica* under salt stress.

Transcription factors (TFs) play a critical role in the transcriptional regulation system and widely participate in the regulation of diverse, crucial cellular activities in plants. A total of 4596 transcription factors were confirmed based on the iTAK software, and RLK-Pelle_DLSV (219), bZIP (125), C3H (125) and MYB-related (119) were the top four TF families (Figure 5 and Supplementary Table S4). These TFs identified from the full-length transcriptome lay a foundation for exploring the molecular regulation mechanism of salt tolerance in *N. sibirica*.

### 3.3. SSR Discovery

**Figure 6:**
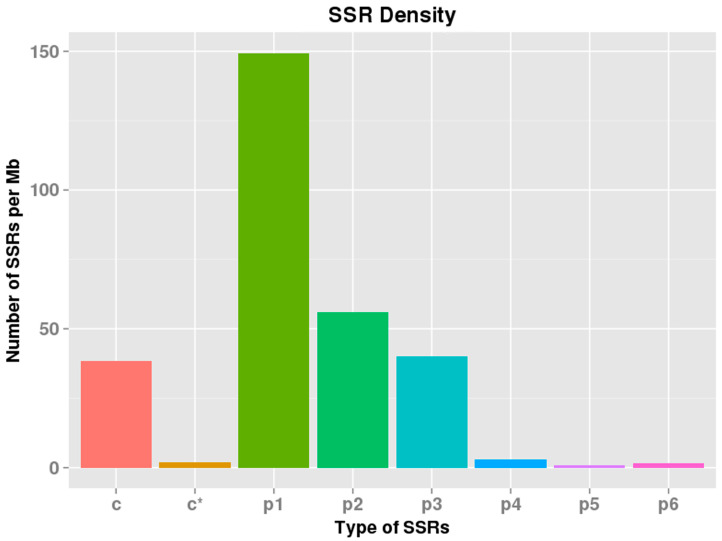
SSR density distribution.

The MIcroSAtellite identification tool (MISA) is software for identifying simple repetitive sequences. In our analysis, the MISA software was used for SSR detection from the full-length transcript data. In total, 37,261 SSR candidates were identified from our full-length transcriptome; the mononucleotide motif (21,885, 58.73%) was the main type of SSR, followed by the dinucleotide motif (9024, 24.21%), trinucleotide (5553, 14.90%), tetranucleotide (466, 1.25%), hexanucleotide motif (209, 0.56%) and pentanucleotide (124, 0.33%) (Table 1). In addition, the SSR density result also showed similar results (Figure 6 and Supplementary Table S5).

### 3.4. LncRNA Prediction and Alternative Splicing Analysis

**Figure 7:**
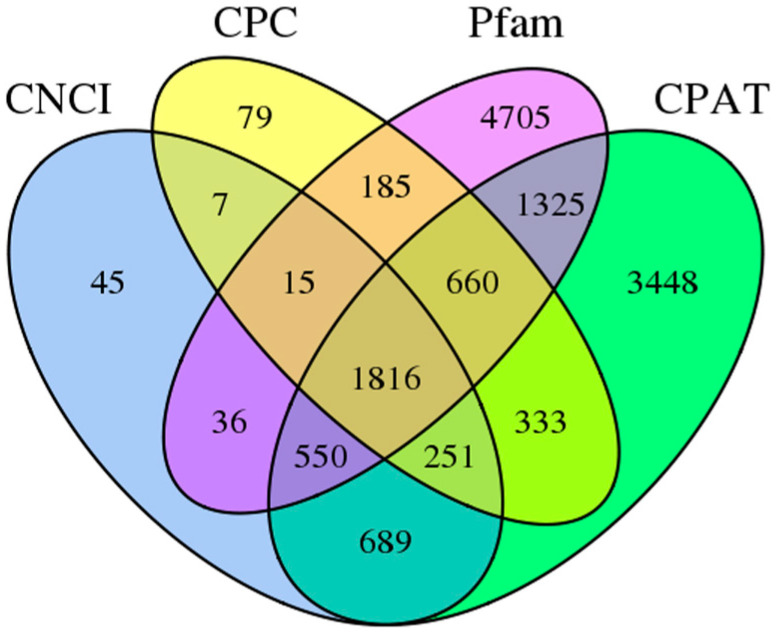
LncRNAs predicted based on CNCI, CPC, Pfam and CPAT.

As lncRNA does not encode protein, by screening the coding potential of the transcript, we can judge whether it has the coding potential, filter out the transcript with the coding potential and then obtain the candidate lncRNA. In the present study, we used four tools to identify the lncRNA from the transcriptome. In addition, we constructed a Venn diagram to describe the lncRNAs screened via the four methods to identify common sequences (Figure 7). The numbers identified by CNCI, CPC, Pfam and CPAT were 3409, 3346, 9292 and 9072, respectively. A total of 1816 common sequences were identified in four tools at the same time (Figure 7). In addition, the target genes of lncRNAs were predicted (Supplementary Table S6). The information of alternatively spliced (AS) isoforms in *N. sibirica* is still unclear. In the present study, candidate AS events were generated from a full-length transcriptome via the all-vs-all BLAST with high identity settings. In our result, there were 2775 candidate AS events that met all the criteria and are considered AS events in *N. sibirica*. 

### 3.5. Functional Annotation

**Figure 8:**
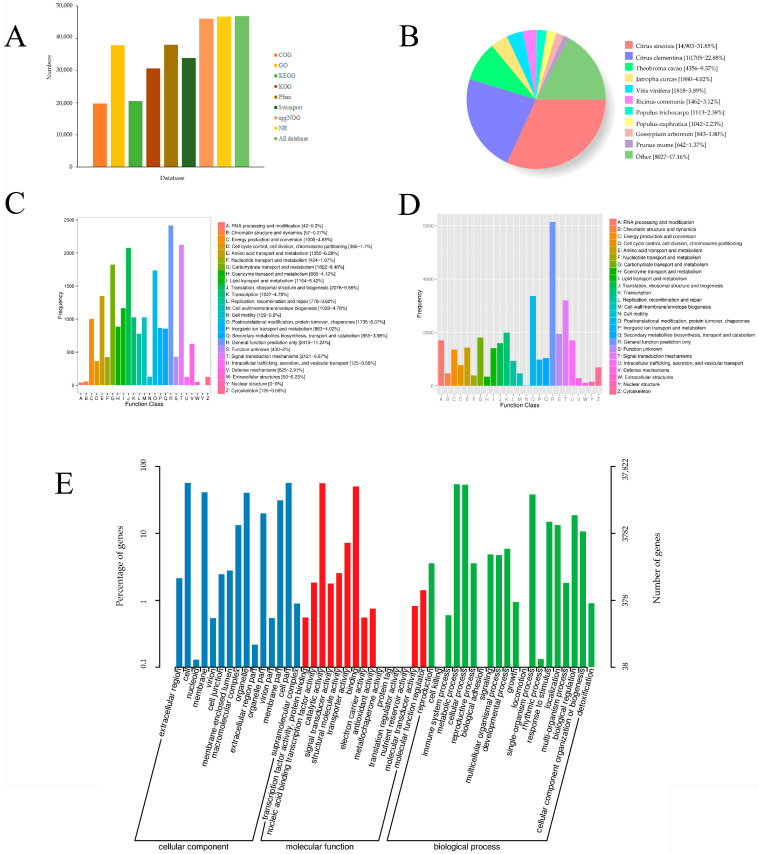
Functional annotation of the corrected transcripts: (**A**) Function annotation of *N. sibirica* transcripts in eight databases. NCBI non-redundant protein sequences database (NR); Protein family (Pfam); a manually annotated and reviewed protein sequence database (Swiss-Prot); Gene Ontology Consortium (GO); Cluster of Orthologous Groups of proteins (COG); Clusters of Orthologous Groups of proteins (KOG); evolutionary genealogy of genes: Non-supervised Orthologous Groups (eggNOG); and Kyoto Encyclopedia of Genes and Genomes (KEGG). (**B**) NR-annotated homologous species distribution. The best hits with an e-value = 10^−6^ for each query were grouped according to species. (**C**) COG annotation of transcript sequences. (**D**) KOG classification diagram of *N. sibirica* transcripts. (**E**) Classification of the all transcripts annotated by the GO.

**Figure 9:**
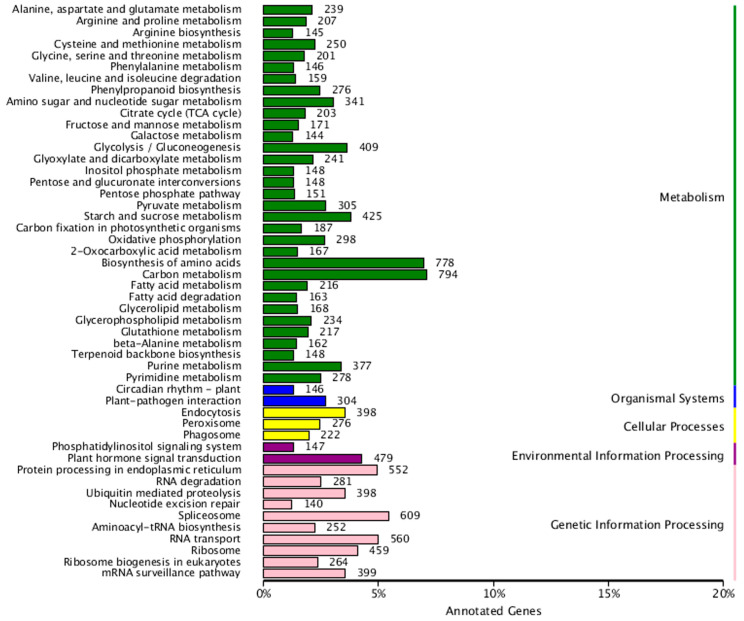
KEGG pathway classification diagram of *N. sibirica* transcripts.

The non-redundant transcript sequences were obtained based on BLAST software (version 2.2.26) and annotated via the COG, GO, KEGG, KOG, Pfam, SwissProt, eggnog and NR databases, and 46,969 transcripts were identified (Figure 8A). The comparison results based on the NR database suggested that 46,799 transcripts were successfully annotated, and sequence similarity with *Citrus sinensis* (14,903) was the highest (Figure 8B). A total of 19,714 and 30,675 transcripts were annotated in the COG and KOG, respectively (Figure 8C,D). There were 20,554 transcripts that were mapped to 129 KEGG pathways and most pathways clustered significantly in the carbon metabolism, biosynthesis of amino acids and spliceosome (Figure 9).

Finally, numerous transcripts in the “cellular component” were mainly associated with the cell, cell part, membrane, organelle and membrane part. The catalytic, binding, transporter, structural molecular activities and nucleic acid binding transcription factor were mainly present in the category “molecular functions”. “Biological processes” such as the metabolic process, cellular process, single-organism process, biological regulation and response to stimulus contain most of the genes (Figure 8E).

### 3.6. Characterization of H^+^-PPase Genes Based on Full-Length Transcriptome

**Figure 10:**
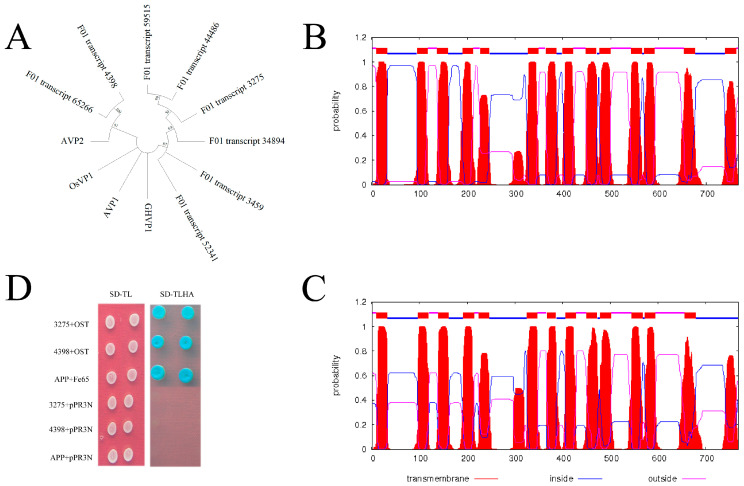
(**A**) Phylogenetic analysis of H^+^-PPase genes. (**B**,**C**) H^+^-PPase transmembrane helix-domain prediction by TMHMM. (**D**) Function analysis by DUAL membrane system, 3275: F01_Transcript_3275-pBT-3N; 4398: F01_Transcript_4398-pBT-3N; OST: positive prey control vector, pOst1-NubI; pPR3N: empty prey vector; Fe65: positive prey vector, pNubG-Fe65; APP: positive bait vector, pTSU2.

With the advantage of SMRT sequencing technology, the coding sequences of H^+^-PPase were identified via the full-length transcriptome data. As a result, eight coding sequences of H^+^-PPase were screened out and named with their coding number in the full-length transcriptome (Supplementary Table S2). Structural analysis showed that all the identified genes contained the Pf03030 domain. The phylogenetic tree constructed by the neighbor-joining method indicated that these genes belonged to the PPase family and were classified into two groups (Figure 10A). After multiple sequence alignment, the two genes (F01_transcript_4398 and F01_transcript_3275) with the longest coding sequences were selected for subsequent analysis. The subcellular location pattern of two candidate genes was predicted based on the TMHMM database. The result showed that F01_transcript_4398 and F01_transcript_3275 were more likely to be localized on the cell membrane (Figure 10B,C). In addition, the predicted TMH numbers of F01_Transcript_3275 and F01_Transcript_4398 were 13 and 14, respectively (Figure 10B,C).

The DUAL membrane system vector, which is a yeast-based screening assay to identify and characterize membrane-associated proteins, was constructed and cotransformed into NMY51 (Figure 10D). The results showed that all cotransformed yeasts grew normally on the TD–LT medium, indicating that the cotransformation was successful. The growth phenotype on the TD–LTHA+α-β-gal plates showed that the F01_transcript_4398 and F01_transcript_3275 grew normally when cotransformed with the positive vector OST (Figure 10D), which indicated that both genes had transmembrane domains and could be used for follow-up experiments. The results of the control group show that our experimental system is reliable (Figure 10D).

## 4. Discussion

*N. sibirica* is a typical halophytic shrub with strong environmental adaptability, which is broadly found in desert, saline–alkali and coastal saline–alkali areas. Therefore, *N. sibirica* is a major landscaping species and native economic tree in these areas. To date, some studies on the salt tolerance and germplasm resources of *N. sibirica* have been carried out [1,2,4,5,6,13,24]. In our previous study, we not only completed the analysis of phenotype and physiology of *N. sibirica* under different salt-stress treatments [1,4,5,7] but also compared the transcriptome data under different salt-treatment concentrations based on NGS sequencing technology [13,27]. We found that the growth of *N. sibirica* was promoted under a 200 mM NaCl concentration but decreased under concentrations higher than 300 mM or without NaCl treatment, while glycophytic plant growth could be seriously damaged under the same conditions [28,29]. That is a specific characteristic of halophyte plants. However, the lack of whole genome information on *N. sibirica* seriously hinders the further development of related molecular research, which is not consistent with its important ecological and economic value. In order to obtain more information on this salt condition, we carried out the full-length transcriptome sequencing based on SMRT technology in this project, aiming to provide more detailed and comprehensive data on this salt-condition characteristic of *N. sibirica* at the molecular level. 

In the present work, we analyzed RNA samples from *N. sibirica* with salt-stress treatments and obtained full-length transcriptome data via the PacBio Iso-Seq platform, which provided more comprehensive genetic information. As a result, sufficient full-length transcriptome data of *N. sibirica* were obtained, including 19.26 Gbp of clean data and 234,508 CCSs, of which 195,027 were screened as FLNC transcripts. Furthermore, based on transcript cluster analysis, 93,713 consensus transcripts were obtained with an average length of 2080 bp, which is significantly better than the average length (555 bp) of transcripts obtained based on the NGS platform in the previous study [27]. This result indicated the advantage of long read-length based on the PacBio Iso-Seq platform, and has also been validated in other species, such as *Sogatella furcifera* (average length 2994 bp) [25], *A. comosus* var. *bracteatus* (average length 2404 bp) [22], and *P. betulifolia* Bunge (average length 1502 bp) [23]. Furthermore, 47,314 coding sequences were generated using TransDecoder sequencing data from the full-length transcriptome data. The number of coding sequences generated by second-generation sequencing was 78,927 [27], which is more than the number in this study. This may be due to the fact that the second-generation sequencing requires the assembly of short reads to obtain the complete sequence, but different assembly methods may not obtain the same results [30]. This result suggests that full-length transcriptome data obtained grounded on SMRT technology may better reflect the complete information about the mRNA in the organism [14,23,31].

LncRNAs are a kind of non-protein coding transcript of longer than 200 nt, which is of great significance in many biological pathways [32,33,34]. Using the non-protein-coding characteristic of lncRNAs, we obtained candidate lncRNAs by filtering out transcripts with coding potential and removing them. At last, 1816 lncRNAs were identified based on CNCI, CPC, Pfam and CPAT methods, and their target genes were also obtained. This number was more than the number in *A. comosus* var. *bracteatus* (329) [22] but less than *P. betulifolia* (7094) [23] and *Ginkgo biloba* L. (4363) [35]. In recent years, the functions of lncRNAs in salt tolerance, such as *Pistacia vera* [36], duckweed [37] and *C. sinensis* [38], were reported. Although crucial progress has been made in the study of lncRNA function in some plants, lncRNA function in *N. sibirica* still needs more studies in the future.

Tissue salt-tolerance is one of the most important strategies for plants to adapt to a saline environment, and one which widely exists in a variety of halophytes. *N. sibirica* is a typical halophyte of the dilute-salt type. Previous studies showed that *N. sibirica* mainly transports Na^+^ into leaves and compartmentalizes it into vacuoles to decrease the toxicity caused by Na^+^ under salt stress [1,4,5]. H^+^-PPase plays a crucial role in the process of vacuolar Na^+^ sequestration, but the molecular network in *N. sibirica* under salt stress remains unclear [1,39,40]. Based on the advantages of the full-length transcriptome, the database obtained by the full-length transcriptome can be used for the screening and identification of functional genes [16,19,31]. In the present work, we identified eight coding sequences of H^+^-PPase from our transcriptome data. The results of subcellular localization showed that the two major H^+^-PPase (F01_transcript_4398 and F01_transcript_3275) had multiple transmembrane domains (Figure 10B,C). Next, their functions were verified based on the DUAL membrane system (Figure 10D), and the results showed that they all had transmembrane regions [40], which is similar to the results of previous studies [40,41]. In addition, the two main H^+^-PPase identified in this study were most closely related to AVP1 in *Arabidopsis*, and F01_transcript_4398 had one more transmembrane structural domain than F01_transcript_3275. This result may imply that the function of H^+^-PPase is redundant in *N. sibirica*, which can help this species to better adapt to the salt habitat. However, further work is needed to reveal the functions of these two H^+^-PPases.

## 5. Conclusions

In the present study, SMRT technology based on the PacBio Iso-Seq sequencing platform was applied to obtain complete and accurate transcriptome data of *N. sibirica*. A total of 37,261 SSRs, 1816 LncRNAs and 47,314 CDSs, of which 40,160 carried complete ORFs, were obtained. These results were easily obtained without reference genome species. In summary, our results show that the SMRT technology based on the PacBio Iso-Seq sequencing platform is a suitable and effective method to obtain accurate and reliable full-length transcripts of *N. sibirica*. Based on our transcriptome data, we also analyzed the coding genes of H^+^-PPase, and the results of both bioinformatics and functional analyses indicated that the gene prediction via full-length transcripts obtained by SMRT technology was complete and accurate. Our data provide a set of reference sequences containing a high-quality and valuable full-length transcript database, which provides useful and valuable support for the further analysis of the regulatory network and molecular mechanism of *N. sibirica* under salt stress.

## Figures and Tables

**Table 1:** SSRs obtained from transcripts.

Search Item	Numbers
Total number of sequences examined	48,442
Total size of examined sequences (bp)	109,925,036
Total number of identified SSRs	37,261
Number of SSR-containing sequences	21,573
Number of sequences containing more than 1 SSR	9024
Number of SSRs present in compound formation	5280
Mononucleotide	21,885
Dinucleotide	9024
Trinucleotide	5553
Tetranucleotide	466
Hexanucleotide	209
Pentanucleotide	124

## Data Availability

The RNA sequencing datasets generated in this study have been deposited in the Sequence Read Archive (SRA) in NCBI under the accession number PRJNA804704. Other data supporting our findings are available in the manuscript file or from the corresponding author upon request.

## Supplementary Materials

The following supporting information can be downloaded at: https://www.mdpi.com/article/10.3390/genes13040661/s1. Table S1: Gene-specific primers used in this study; Table S2: Sequence information of all identified H^+^-PPases; Table S3: The predicted information of CDS sequences; Table S4: The predicted information of TF annotation and sequence; Table S5: The predicted information of SSR; Table S6: The predicted information of lncRNA and their target genes.
